# Selective Review of Neuroimaging Findings in Youth at Clinical High Risk for Psychosis: On the Path to Biomarkers for Conversion

**DOI:** 10.3389/fpsyt.2020.567534

**Published:** 2020-09-23

**Authors:** Justin K. Ellis, Elaine F. Walker, David R. Goldsmith

**Affiliations:** ^1^Department of Psychiatry and Behavioral Sciences, Emory University School of Medicine, Atlanta, GA, United States; ^2^Department of Psychology, Emory University, Atlanta, GA, United States

**Keywords:** schizophrenia, psychosis, clinical high risk, prodrome, neuroimaging, MRI, PET

## Abstract

First episode psychosis (FEP), and subsequent diagnosis of schizophrenia or schizoaffective disorder, predominantly occurs during late adolescence, is accompanied by a significant decline in function and represents a traumatic experience for patients and families alike. Prior to first episode psychosis, most patients experience a prodromal period of 1–2 years, during which symptoms first appear and then progress. During that time period, subjects are referred to as being at Clinical High Risk (CHR), as a prodromal period can only be designated in hindsight in those who convert. The clinical high-risk period represents a critical window during which interventions may be targeted to slow or prevent conversion to psychosis. However, only one third of subjects at clinical high risk will convert to psychosis and receive a formal diagnosis of a primary psychotic disorder. Therefore, in order for targeted interventions to be developed and applied, predicting who among this population will convert is of critical importance. To date, a variety of neuroimaging modalities have identified numerous differences between CHR subjects and healthy controls. However, complicating attempts at predicting conversion are increasingly recognized co-morbidities, such as major depressive disorder, in a significant number of CHR subjects. The result of this is that phenotypes discovered between CHR subjects and healthy controls are likely non-specific to psychosis and generalized for major mental illness. In this paper, we selectively review evidence for neuroimaging phenotypes in CHR subjects who later converted to psychosis. We then evaluate the recent landscape of machine learning as it relates to neuroimaging phenotypes in predicting conversion to psychosis.

## Introduction

Schizophrenia is a debilitating illness that affects 1% of the global population (1, 2), shortens the lifespan of those afflicted (3), and imposes a substantial financial burden on patients, their families, and society (4, 5). Clinically, it is characterized by positive symptoms, such as hallucinations and delusions, negative symptoms, such as anhedonia and amotivation, and cognitive symptoms, such as deficits in working memory, executive function, and attention. Despite significant ongoing efforts to understand the pathophysiology of this disease, currently available treatments are generally only successful in ameliorating the positive symptoms. However, it is the negative and cognitive symptom burden that correlate most with overall decline in global functioning and lifespan (6, 7), and no adequate treatments currently exist. Thus, more and more efforts have begun to look at early identification of illness, with the goals of predicting disease onset and severity, and ultimately, prevention of conversion to first episode psychosis.

Diagnosis of schizophrenia usually occurs in late adolescence with the onset of a first psychotic episode. Prior to a first episode of psychosis (FEP), patients experience a prodromal period of 1–2 years, during which symptoms of psychosis first appear in an attenuated form and then progress. Prodromal symptoms are also characterized by social withdrawal, increased isolation, and a global decline in functioning (8–10). During that time period, subjects are referred to as being at Clinical High Risk (CHR), as a prodromal period can only be designated in hindsight in those who convert. 30–35% of clinical high risk subjects will experience a first psychotic episode and be diagnosed with a primary psychotic disorder (11, 12). Of those who do not, approximately 7% will recover, 28% will continue to experience persistent, attenuated psychotic symptoms, and 65% will be diagnosed with another non-psychotic psychiatric disorder (12). The clinical high risk period represents a critical window during which targeted interventions may be developed and applied. Therefore, predicting who among the this population will convert is of critical importance.

For the past 100 years, neuroimaging has taken a distinguished role in providing new insights into the pathophysiology of schizophrenia and is uniquely primed to evaluate the adolescent brain both pre- and post-first psychotic episode. To date, a variety of neuroimaging modalities have identified numerous differences between CHR subjects and healthy controls. However, thus far the majority of studies have been cross-sectional in design, and a significant degree of variation among phenotypes have been reported. Further complicating attempts at predicting conversion is the increasingly recognized co-morbidity of other psychiatric diagnoses among CHR subjects. In one study, 79% of CHR subjects met criteria for comorbid psychiatric diagnoses, including mood, anxiety, and substance use disorders (13). In a follow up report, 60% of CHR subjects were diagnosed with comorbid major depressive disorder, which was associated with more pronounced negative and general symptoms, as well as poorer prognosis (14). Comorbidity, thus far, has not been associated with conversion to psychosis. Nevertheless, it has become quite clear that phenotypes discovered between CHR subjects and healthy controls are likely non-specific to psychosis and generalized for major mental illness. In order to improve prediction algorithms there needs to be a greater focus on longitudinal studies that identify phenotypes present among converters and non-converters.

In this narrative review, we selectively evaluate evidence for neuroimaging phenotypes in CHR subjects who later converted to psychosis. We then evaluate the recent landscape of machine learning and prediction algorithms as they relate to neuroimaging phenotypes in predicting conversion to psychosis.

## Structural Phenotypes

### Enlarged Ventricles

The first report of enlarged ventricles in patients with schizophrenia was in 1927 using pneumoencephalography (PEG) to measure ventricular size (15). Despite early concerns due to lack of controls and variation in methodology, this observation is one of the most replicated findings in the literature using both computed tomography (16) and magnetic resonance imaging (17–19). Originally studied in chronic cases, ventricular enlargement has been observed and well replicated in first episode psychosis. In support of this, three meta-analyses have reported ventricular enlargement in FEP patients (20–22). All three found enlargement in the lateral ventricles compared to controls, but two also observed enlargement of the 3^rd^ ventricle (21, 22). The 3^rd^ ventricle was not measured in the third meta-analysis (20).

As ventricular enlargement is such a consistent finding in FEP patients, it is surprising that few studies have investigated ventricular enlargement in the CHR population. To the best of our knowledge, there are only two longitudinal studies evaluating ventricular size in converters versus non-converters, and there are discrepancies in their findings. Ziermans et al. evaluated 43 CHR subjects, 8 of whom converted to psychosis, and found no difference in lateral ventricular volume among converters and non-convertors in post-hoc analysis (23). 3^rd^ ventricular volume was not measured. However, in a much larger study, Cannon et al. evaluated 274 CHR subjects, of whom 35 converted to psychosis (24). They did not observe enlarged lateral ventricles, but did observe expansion of the 3^rd^ ventricle in CHR subjects who converted to psychosis compared to both non-convertors and controls. Furthermore, a shorter prodromal period before conversion was associated with greater expansion of the ventricle.

Although not many studies appear to have looked specifically at ventricular enlargement in CHR subjects, those that did failed to find enlargement in the lateral ventricles at baseline. However, one phenotype that warrants further investigation and replication is enlargement of the 3^rd^ ventricle in CHR subjects at baseline that later convert to psychosis.

### Decreased Grey Matter Volume

Reductions in grey matter volume in multiple brain regions have been well established in patients with schizophrenia (25). In FEP, multiple meta-analyses have reported whole brain reductions in grey matter volume (20–22), as well as reductions in hippocampal volume. Specifically, anterior hippocampal volume deficits have been reported in FEP (26), with “anterior” defined as containing the CA1, CA3, CA4, molecular layer, GC/DG, and subiculum/presubiculum subfields. Deceases in grey matter volume are also clinically relevant as they are positively correlated with symptom severity (27). Furthermore, degree of grey matter loss in the cerebellum within the first year of diagnosis has been correlated with worsening of negative symptoms and functional outcome at 5 year follow up (28).

To our knowledge, there is thus far only two reports that examined whole brain grey matter volume in CHR subjects. One reported a reduction in whole brain grey matter volume, (29), but the other did not (30), although the study may have been underpowered. However, multiple subsequent studies in CHR subjects have identified individual brain regions exhibiting grey matter reduction both at baseline compared to controls and post-conversion to psychosis. Two meta-analyses by the same group revealed that subjects who converted to psychosis had baseline reductions in the right inferior frontal gyrus and the right superior temporal gyrus compared to non-converters (31, 32). Although they didn’t follow subjects longitudinally, Iwashiro et al. reported bilateral reduction of the pars triangularis within the inferior frontal gyrus in CHR subjects, and the degree of reduction was negatively correlated with severity of positive symptoms (33). Another large study reported reduced grey matter volume in the left parahippocampal cortex in CHR convertors compared to non-convertors (34). Increased grey matter loss in the right superior frontal, middle frontal and medial orbitofrontal regions was reported in CHR subjects who converted compared to both non-convertors and healthy controls (24). Grey matter loss occurred in the absence of treatment with antipsychotics, and reduction was also steeper in convertors who exhibited shorter duration of prodromal symptoms. An adjunct study to the previous report found a positive correlation between severity of prodromal symptoms, especially unusual thought content, and degree of grey matter loss among converters (35). Decrease in the right prefrontal region (36) and the right insular cortex (37) has been observed in convertors compared to non-convertors. Degree of decrease in the prefrontal region was associated with more severe negative symptoms at baseline, and longitudinally, convertors showed greater reduction over time compared to non-convertors. Finally, decreased grey matter in the right medial temporal, lateral temporal and inferior frontal cortex, and cingulate cortex bilaterally was observed at baseline in those who in converted compared to non-convertors (38). Collectively, these studies consistently identify grey matter deficits in the prefrontal cortex cingulate cortex and temporal lobes in CHR subjects who convert to psychosis versus those who do not, indicating that deficits in these regions may be more specific to psychosis than generalized mental illness.

### White Matter Deficits

Although grey matter deficits have received much of the focus of investigation, multiple observations of white matter disruption in patients with schizophrenia have been reported (39). Postmortem data has revealed abnormal numbers and morphology of oligodendrocytes (40, 41). Genome wide association studies have also shown an increase in risk related to single nucleotide polymorphisms in oligodendrocyte specific genes (42). Furthermore, rodent models have shown that 2^nd^ trimester insults, especially maternal infection, a known risk factor for schizophrenia (43), can produce a decrease in fractional anisotropy (FA) in fronto-striatal-limbic circuitry similar to that seen in the illness (44). Supporting these discoveries, investigators have characterized white matter abnormalities in the CHR population.

Voxel based morphometry of structural magnetic resonance images has been used to investigate white matter volume in CHR subjects. In a cross-sectional study, Witthaus et al. reported a reduction in white matter volume in the right superior temporal lobe in CHR subjects compared to controls. This observation was enhanced in a separate cohort of FEP patients but not studied longitudinally in order to compare converters vs non-converters (45). However, imaging of the anterior genu of the corpus callosum revealed a significant reduction in thickness in CHR subjects who later converted to psychosis compared to both controls and CHR subjects who did not convert (46). Furthermore, the authors reported that a Cox regression analysis revealed that mean anterior genu thickness was predictive of transition to psychosis.

Diffusion tensor imaging, which indirectly measures the integrity of white matter tracts based on the diffusion of water molecules, has also been used to evaluate white matter integrity in CHR subjects. Unfortunately, to date, most studies did not follow CHR subjects longitudinally to evaluate baseline differences in converters vs non-converters. Furthermore, the findings are heterogeneous. Reduced fractional anisotropy has been reported both globally (47), as well as in the cingulum bundle (48), in cross sectional studies of CHR subjects at baseline compared to healthy controls. Furthermore, Karlsgodt et al. observed reduced FA in the superior longitudinal fasciculus (SLF) (49) in a similar comparison of CHR subjects to controls, and the SLF was also reported to exhibit increased mean diffusivity, another measure of reduced white matter integrity, in a different study (50). In a longitudinal study of CHR subjects that converted to psychosis, decreased FA was observed in the left frontal lobe (51). Bloemen et al. reported a similar finding; decreased FA in the bilateral medial frontal lobes, as well as the left putamen and the left superior temporal lobe in CHR subjects who converted compared to non-convertors and controls (52). However, not all investigations have yielded positive results. Peters et al. evaluated the uncinate and arcuate fasciculi, the anterior and dorsal cingulate, and subdivisions of the corpus callosum and did not find any differences between CHR subjects who converted to psychosis and those who did not (53).

Overall, decreased thickness in the corpus callosum and decreased FA in the frontal and temporal lobes are the most consistent phenotypes in convertors to psychosis. However, these findings require further replication in larger sample sizes.

## Functional Phenotypes

### Regional Abnormalities

With the development of fMRI, researchers were able to move beyond structural abnormalities and begin inferring changes in cortical activity *via* localized changes in cerebral blood flow and neurovascular coupling, either at rest or during specific cognitive tasks, in relevant brain regions for schizophrenia. One of the earliest and most consistent findings has been hippocampal hyperactivity at baseline in patients with chronic disease (54). The same finding was observed in first episode psychosis, as well as decreased recruitment during a scene processing task compared to controls (55). Interestingly, the degree of recruitment was inversely correlated with baseline activity. The authors attributed these findings to a worsening imbalance in excitation/inhibition as a result of interneuron dysfunction. To evaluate hippocampal activity in CHR subjects, arterial spin labeling (ASL) was used to measure regional cerebral blood flow (rCBF) (56). CHR subjects exhibited increased rCBF in the hippocampus, as well as in the basal ganglia and midbrain. Furthermore, subjects whose symptoms improved and no longer met criteria for CHR exhibited a significant reduction in left hippocampal rCBF. Unfortunately, subjects were not followed for progression to psychosis.

Multimodal imaging has been used to evaluate relationships between hippocampal activity and other neurotransmitters in CHR subjects. GABA concentration in the medial prefrontal cortex (mPFC) was measured using magnetic resonance spectroscopy (MRS), and a positive correlation was detected with hippocampal rCBF in subjects who converted to psychosis compared to non-convertors (57). MRI, fMRI, and MRS were combined to measure grey matter volume, cerebral blood volume (CBV), and glutamate in the hippocampus of CHR subjects, and both elevated glutamate and CBV was observed compared to controls (58). However, only baseline hippocampal atrophy predicted conversion to psychosis.

Deficits in working memory and the dorsolateral prefrontal cortex (DLPFC) have long been reported in patients with schizophrenia (59, 60). To evaluate DLPFC recruitment during working memory tasks, CHR subjects performed an item recognition task at baseline and were then followed for 2 years for conversion (61). CHR subjects performed as well as controls during the task. However, CHR subjects who later converted to psychosis showed a positive association between age and greater activation of the DLPFC, inferior frontal gyrus, frontal eye fields, and superior frontal gyrus, during verbal working memory tasks. The authors speculate that the greater activation may reflect compensatory activity. In CHR subjects who did not convert, several regions were positively associated with age and greater activation, but they were diffusely spread out throughout the temporal, parietal and occipital lobes, and not in the frontal lobes. Control subjects showed a negative association with age and activation of the DLPFC during verbal WM tasks, which was hypothesized to reflect maturation, and thus, greater efficiency of the circuit. In a different working memory task, the superior temporal gyrus (STG) showed reduced activation in controls, greater activation in subjects with FEP, and an intermediate level of activation in CHR subjects (62). The STG also failed to de-couple with the middle frontal gyrus, a finding that was even more pronounced in FEP subjects. Finally, CHR subjects showed decreased activation in fronto-parietal regions during encoding of a working memory task (63), along with increased activation in the STG.

### Network Abnormalities

The Default Mode Network (DMN) is an interconnected set of brain regions, consisting of the mPFC, the posterior cingulate cortex, the inferior parietal lobules, the precuneus, and the medial temporal lobes. Functionally, the DMN is thought to be involved in internal mentation, such as thoughts regarding one’s self, thoughts about others, and reflecting on the past. Of particular importance, multiple regions of the DMN exhibit significant grey matter volume loss in patients with schizophrenia. Therefore, it is unsurprising that functional DMN abnormalities have been reported. In patients with schizophrenia, increased activity at rest is routinely observed compared to controls, and the degree of increase correlates to the severity of positive symptoms (64, 65).

CHR subjects also exhibit functional abnormalities in the DMN, although, to date, very few studies have investigated differences between converters and non-converters. In a verbal working memory task, healthy controls exhibited load dependent decreases in DMN activity, whereas CHR subjects maintained inappropriately elevated levels of DMN activity (66). CHR deficits were similar to, but less pronounced than, those seen in FEP subjects. Increased DMN connectivity, between the PCC/Precuneus and vmPFC, in CHR subjects is also associated with poorer clinical insight (67). Furthermore, graph theoretical analysis revealed a progressive reduction in efficiency in the DMN and an increase in network diversity in subjects who converted to psychosis (68), indicating continuing changes in brain networks as psychosis develops. Increased cerebellar-default mode network connectivity was also reported at resting state in CHR subjects (69). Specifically, there was increased connectivity between the right Crus 1 of the cerebellum and bilateral PCC/precuneus and between Lobule IX of the cerebellum and the left superior medial prefrontal cortex. There was also a positive correlation between precuneus connectivity and SIPS and PANSS scores in CHR subjects.

Patients with chronic disease have also been shown to exhibit functional dysconnectivity between the ventrolateral prefrontal cortex (vlPFC) and the amygdala (70). To evaluate this relationship prior to illness onset, CHR subjects were given an emotion activation task, and functional connectivity between the vlPFC and amygdala was evaluated (71). While performing the task, CHR individuals exhibited a proportional increase in activation in the amygdala and decrease in activation of the vlPFC, whereas controls exhibited the opposite pattern.

Another highly reproduced finding in the CHR population is disruptions in thalamocortical connectivity. Thalamocortical connectivity is disrupted at baseline in CHR subjects, and even more so in those who convert to psychosis (72). Specifically, there is hypoconnectivity between the thalamus and the prefrontal cortex, as well as the cerebellum. Furthermore, there is hyperconnectivity between the thalamus and the sensory motor areas. A meta-analysis on thalamocortical connectivity at baseline in CHR subjects found hypoconnectivity between the thalamus and the middle frontal and cingulate regions (73). Hyperconnectivity was found in motor, somatosensory, temporal, occipital, and insular regions. Furthermore, a strong negative correlation was found between hypo and hyperconnectivity, indicating that abnormalities in one are likely influencing abnormalities in the other. Finally, hyperconnectivity in the cerebello-thalamo-cortical circuitry has been reported, which correlated with degree of disorganized symptoms and time to conversion (74). The finding was also observed in patients with chronic schizophrenia.

Together these studies indicate multiple focal and regional abnormalities in functional connectivity in CHR subjects, some of which seem to be specific to conversion to psychosis. Further studies, specifically looking at conversion, are needed to validate some of the more promising phenotypes, such as baseline hippocampal activation and thalamocortical dysconnectivity.

## Inflammatory Phenotypes

Inflammation has long been associated with the pathophysiology of schizophrenia (75). Winter births and maternal infections (43), genetic risk associated with the major histocompatibility complex (76), and subsequent discovery of the association of complement protein C4A (77) all represent converging evidence for the involvement of inflammation in the disease. Furthermore, in CHR subjects, several lines of evidence indicate increased inflammation prior to first episode psychosis. Increased peripheral cytokines have been associated with both symptom severity and degree of grey matter loss in CHR subjects (24), as well as predicting conversion to psychosis (78), and peripheral TNF-alpha levels have been shown to predict negative symptom severity (79).

Translocator Protein 18D (TSPO) is an outer mitochondrial membrane protein with multiple functions that is found throughout the body. Increased expression in the brain has been linked to injury from any etiology (80), as well as activation of both microglia and astrocytes (81, 82). Thus, investigators have used PET imaging to measure degrees of activation and try to extrapolate levels of inflammation in the brains of patients with schizophrenia. Furthermore, given the hypothesis that grey matter loss may be secondary to hyperactive microglia, it was thought that elevated TSPO might be an indicator of this activity. Early studies in chronic cases reported an increase in TSPO signal in both total grey matter (83) and in the hippocampus (84). Subsequently, investigators began looking at CHR subjects for evidence of microglial activation prior to FEP. Of note, multiple radiotracers have been used to measure TSPO activation in the brain *via* PET imaging. [^11^C]PK11195 was the first to be widely utilized. However, due to the relative non-specific binding of [^11^C]PK11195, 2^nd^ generation radiotracers were developed with significantly higher binding affinity; [^11^C]DAA1106, [^18^F]FEPPA, and [^11^C]PBR28. However, due to the rs6971 polymorphism in the TSPO gene, a subject may be a high, medium, or low-affinity binder of the newer radiotracers. Therefore, genotyping of subjects prior to inclusion in a study, which is not always performed, is essential for accurate data interpretation. Complicating matters further, more recent studies using the 2^nd^ generation ligands have failed to show an increase in TSPO in chronic disease (85, 86), and one meta-analysis (87) concluded that there was a decrease in TSPO signal.

Evaluating multiple cortical and subcortical brain regions, no evidence of increased TSPO signal was reported in CHR subjects using [^11^C]PK11195 as the radioligand (88). Using the ligand [^18^F]FEPPA in CHR subjects, and controlling for the TSPO rs6971 polymorphism, no differences were observed in either the DLPFC or the hippocampus (89). Operating under the hypothesis that microglial pruning may be causative in grey matter loss, the same group then attempted to correlate changes in TSPO with grey matter volume reductions in CHR subjects. They found a positive correlation between increased TSPO signal and grey matter volume loss in FEP, but not in CHR subjects (90). Selvaraj et al. used the [^11^C]PBR28 ligand to investigate the same relationship and also failed to observe an association between cortical grey matter volumes and TSPO signal in CHR subjects (91). They did find a negative association in patients with schizophrenia, suggesting that TSPO may be related to grey matter loss as the disease progresses. One positive finding has been reported. Using [^11^C]PBR28, TSPO signal was elevated in total grey matter in CHR subjects at baseline compared to controls and was positively correlated with symptom severity (92). Patients with schizophrenia exhibited the same finding. Unfortunately, subjects were not followed longitudinally to evaluate signal changes in those who converted.

Beyond measuring TSPO signal levels in isolation, other groups have combined PET imaging with magnetic resonance spectroscopy (MRS) in order to examine the relationship between TSPO and other molecules. A negative correlation was reported between glutathione levels, an anti-oxidant, and TSPO using [^18^F]FEPPA, in the medial prefrontal cortex (mPFC) of healthy volunteers (93). However, this association was not present in CHR subjects, suggesting an abnormal redox status in this population. No differences were seen in TSPO or glutathione levels between groups in direct comparisons. Also in the medial mPFC, a region highly implicated in the disease, GABA levels were negatively associated with TSPO signal in CHR subjects (94). Finally, PET imaging was used to measure dopamine release in the prefrontal cortex (PFC) during a stress task in CHR subjects. Subjects with lower stress induced PFC dopamine release exhibited higher TSPO increase in the hippocampus (95).

Although the findings involving TSPO signal and schizophrenia have been heterogeneous and controversial, no studies have yet examined TSPO signal between CHR subjects that converted to psychosis and those that did not. Given the growing evidence for the involvement of inflammation, it may be prudent to perform these experiments before closing the door on this modality.

## Neurotransmitter Specific Phenotypes

Positron Emission Tomography (PET) is a common imaging modality that has been used to study the dynamics of neurotransmitter synthesis and release in patients with schizophrenia. Using radiotracers, such as 3,4-dihydroxy-6-[^18^F]fluoro-L-phenylalanine (^18^F-DOPA), researchers have been able to establish that aberrations in neurotransmitter systems, such as the dopaminergic system, are common in patients with chronic disease (96). Abnormalities have been found in presynaptic dopamine synthesis (97, 98), dopamine release following amphetamine administration (99, 100), and in occupancy of D2 receptors (101, 102). PET is now being used to examine neurotransmitter systems in the CHR population to investigate if similar abnormalities are present prior to FEP.

Increased ^18^F-DOPA uptake was reported in the striatum, specifically the associative subdivision, of CHR subjects (103), indicating increased dopamine synthesis capacity, a finding that was replicated in a second cohort (104). Clinical follow up of the first cohort revealed that CHR subjects with the highest level of striatal dopamine synthesis converted to psychosis (105), and that progression towards psychosis was associated with increasing levels of dopamine (106). Other groups have also found increased fluorodopa uptake in the associative striatum in CHR subjects (107). Increased ^18^F-DOPA uptake has been reported in the midbrain region in CHR subjects who converted compared to non-convertors (108). ^1^H-MRS was used to measure hippocampal glutamate activity and was combined with ^18^F-DOPA dopamine synthesis capacity in the evaluation of CHR subjects (109). Striatal dopamine synthesis capacity predicted worsening psychotic symptoms at clinical follow up, but not transition to psychosis, and was not significantly related to hippocampal glutamate concentration.

Recently, investigators have begun combining fMRI with PET imaging in order to correlate activation of implicated brain regions with neurotransmitter dysfunction. When given a verbal encoding and recognition task, CHR subjects showed a positive correlation between medial temporal lobe activation and striatal dopamine synthesis during encoding but not recognition (110). When given the Salience Attribution Test, CHR subjects were more likely to attribute motivational salience to irrelevant stimuli, and dopamine synthesis capacity was negatively correlated with hippocampal responses to irrelevant stimuli (111). Magnetic Resonance Spectroscopy (MRS) was used measure baseline hippocampal glutamate levels in CHR subjects, and higher levels were recorded in subjects who converted to psychosis (112). Higher levels were also associated with a poor functional outcome.

## Machine Learning and Prediction Algorithms

The first part of this review summarized neural imaging phenotypes observed in CHR subjects, with an emphasis on subjects that converted to first episode psychosis compared to subjects who did not. The second part of this review will discuss the significant efforts that have been made using machine learning approaches to translate those observations into clinically relevant classification and prediction algorithms. As discussed in previous sections, a significant number of neuroimaging phenotypes have been discovered that differentiate CHR subjects who converted from those who did not. However, most of those studies evaluated average differences at the group level, which do not allow for inference or prediction at the individual level. With advances in computational methods, the field could move forward from traditional neuroimaging analytic approaches to more sophisticated methodology that would employ neuroimaging data to make clinically relevant diagnoses and predictions. Machine learning, an application of artificial intelligence, allows for multivariate analyses and pattern recognition, which then allows for inference at the individual level. There are multiple machine learning methods, but the most common type applied to neuroimaging data in psychiatry has been the support vector machine (SVM). An SVM is a form of supervised learning, which learns by being trained on an initial dataset of known outcome and is then validated by applying it to another independent data set of known outcome [for further review of SVM and neuroimaging datasets see Orru et al., Neurosci Biobehav Rev, 2012, ref (113)]. In the realm of CHR subjects and psychosis, SVM has been used both in the classification and diagnosis of CHR subjects, as well as prediction of conversion to psychosis.

### Machine Learning and Clinical Phenotypes

The first attempts at creating and validating risk calculators for conversion to psychosis were based solely on clinical symptomatology. From these early studies (11, 114), several high risk symptoms were able to be identified, such as high unusual thought content score, social impairment, and genetic risk for schizophrenia plus recent functional decline, and were part of one of the 1^st^ psychosis risk calculators (115). The calculator achieved a C-index, similar to AUC but applicable to censored data, of 0.71, with a sensitivity and specificity of 66.7 and 72.1%, respectively, which indicates fair predictive accuracy. Risk calculators using similar variables were also created in China (116) and the UK (117) with equivalent results. However, the early risk calculators were based on inferences at the group level, making the applicability to the individual unclear. The first study to apply machine learning and SVM to clinical variables to predict individual transition to psychosis came from the PACE clinic in Australia. Four hundred sixteen subjects were included, and the accuracy of individual prediction was 64.6% with reported sensitivity and specificity of 68.6 and 60.6%, respectively (118). For an excellent table summarizing studies of clinical predictors of conversion to psychosis, see Worthington et al., Biol Psych, 2020, ref (119).

These early pioneering studies were useful in identifying which symptoms represent the greatest risk for conversion and showing the applicability of using machine learning to make predictions at the individual level. However, one inescapable conclusion from these studies is that while progress has been made in using machine learning to expand the predictive capabilities of risk calculators, clinical and demographic variables alone cannot predict individualized risk for conversion with a high enough accuracy to be clinically relevant. As discussed below, a combination of modalities and phenotypes will likely be necessary.

### Machine Learning and Neuroimaging Phenotypes

Building upon the neuroimaging phenotypes between in CHR subjects, investigators have built machine learning algorithms to classify CHR subjects based on neuroimaging scans using structural and functional data sets. For example, Bendfeldt et al., evaluated fMRI data during a verbal working memory task from 19 CHR subjects and 19 controls and were able to separate CHR subjects from Controls with a balanced accuracy of 76.2% (sensitivity 89.5% and specificity 63.2%) (120). However, their algorithm could not correctly classify CHR from FEP or FEP from controls, likely due to small sample size. Another fMRI study of 34 CHR subjects and 37 controls focused on regional homogeneity, which summarizes functional connectivity between a given region and its local neighboring regions, and was able to classify CHR subjects with a sensitivity and specificity of 88 and 91%, respectively (121). In doing so, they noted that CHR subjects exhibited significant decreases in regional homogeneity in the left inferior temporal gyrus and increases in the right inferior frontal gyrus and right putamen compared with the controls. Of importance, Salvador et al. attempted to use structural MRI and a wide range of machine learning methods, as well as multiple structural metrics, to classify schizophrenia subjects versus controls (122). However, the largest balanced accuracy did not exceed 75%. Furthermore, their sample size of 128 patients with schizophrenia and 127 controls was considerably larger than the two previous studies. These results imply that, like clinical predictors, neuroimaging datasets alone may not be enough to achieve a level of accuracy necessary to be clinically relevant. One way investigators have sought to increase classification accuracy is by applying machine learning to multimodal datasets. For example, Valli et al. utilized machine learning to classify CHR subjects from controls by combining univariate and multivariate analyses to look at structural MRI and functional MRI during a verbal memory task (123). SVM applied to the structural MRI datasets identified CHR subjects from Controls with an accuracy of 72% (sensitivity and specificity of 68 and 76%, respectively). They also identified univariate differences at the group level in the fMRI data in the left middle frontal and precentral gyri, supramarginal gyrus, and insula as well as the right medial frontal gyrus. Finally, Lei et al. used SVM to analyze structural MRI datasets of both grey and white matter and rs-fMRI to classify schizophrenia vs controls and obtained an accuracy of 90.83% (124). The study utilized a multi-site design, which resulted in 295 patients and 452 controls at 5 different sites. Of note, they analyzed the datasets collected at each site separately because the SVM algorithm created for each dataset did not perform well when applied to the datasets at the other sites, a phenomenon that will be further discussed below.

Two other studies used machine learning to discover new phenotypes in the classification of CHR subjects. Chung et al. trained a machine learning algorithm on grey matter volumes in healthy subjects and correlated those measurements to subjects’ chronological age to create a “brain age” (125). They then applied their algorithm to structural MRI scans from 275 CHR subjects. The difference between the estimated brain age and the chronological age was termed the “brain age gap”. Overall, CHR subjects exhibited a brain age gap of 0.64[2.16] years. Younger CHR subjects (12–17 years) who later converted exhibited a brain age gap of 1.59 years. Furthermore, the top 25 (out of 92) brain regions studied aligned with areas of significance to schizophrenia. A similar study used cognitive measures to create an algorithm to predict “neurocognitive age” relative to chronological age, and found that CHR subjects have delayed neurocognitive maturation of approximately 4.3 years compared to controls (126). However, this did not differ in converters vs non-converters. These studies show how machine learning can be used to generate new phenotypes that may aid in both classification and prediction.

Only a few studies to date have used machine learning algorithms to predict conversion to psychosis among CHR subjects. In 2012, Koutsouleris et al. trained an SVM algorithm on structural MRI datasets among 37 CHR subjects (16 of whom converted) and 22 volunteers (127). A balanced accuracy of 84.2% was achieved in classifying converters vs non-converters (sensitivity 81%, specificity 87.5%). A follow up study by the same group validated their previous findings in 73 CHR subjects from two different sites (128). This time, the accuracy of prediction was 80% (sensitivity 76%, specificity 85%). They also used their algorithm to stratify subjects at baseline into high, intermediate, and low risk, and the high-risk group had a transition rate of 88% and the low risk group had a transition rate of 8%.

One complication in predicting conversion to psychosis is that there are potentially multiple pathophysiological routes. As a result, being able to predict functional outcome, regardless of presence or absence of psychosis, may be just as valuable. Several investigators have used machine learning to explore this avenue. Kambietz-Ilankovic et al. used structural MRI at baseline and the Global Assessment of Functioning (GAF) scale at clinical follow up to predict functional outcome in 27 CHR subjects (129). Classifying outcome as “good” or “poor” achieved an accuracy of 82%. In a similar vein, de Wit et al. looked at predicting resilience as a primary outcome in 64 CHR subjects (130). They, as well, used sMRI at baseline and the GAF score at 6 year clinical follow up as an indicator of resilience. However, they used support vector regression analyses, allowing for predictions along a continuous, instead of binary, scale. The highest correlation, 0.42, was found between long term functioning and subcortical volumes. Finally, a report by the PRONIA consortium combined clinical variables with structural MRI datasets to predict 1 year social and role-functioning outcome in 116 CHR subjects (131). The accuracy of prediction using clinical variables was 76.9%, using structural MRI variables was 76.2%, and in combined models was 82.7%. These results show definitively how combining multi-model datasets increases accuracy of prediction and will be necessary moving forward.

To summarize, the application of machine learning to neuroimaging datasets has allowed for new paradigms to be created in the classification and outcome prediction of CHR subjects. However, it is clear that a single modality, whether clinical, imaging, or other, will likely not provide enough information to allow for more accurate predictions. A combination of clinical variables and neuroimaging data improves prediction accuracy compared to either modality alone. Continued application and testing of different modalities in different combinations will be essential.

## Discussion and Conclusion

The prodromal period in schizophrenia, during which time clinically high-risk subjects experiencing attenuated symptoms may present for care, represents a critical window for identification, stratification of risk, and implementation of appropriate therapies. Although the illness carries a strong genetic risk, the leading theory surrounding development of schizophrenia is the “two hit” phenomena, whereby environmental stressors act upon genetic predisposition to initiate progression to first episode psychosis. This implies that development of illness may not be inevitable, and that prevention of conversion is not an unreasonable goal. For this to occur, however, progress needs to continue in several areas. There must be continued identification of biomarkers in longitudinal studies that follow CHR subjects through conversion. Only then will it be possible to segregate abnormalities at baseline into genetic or clinical risk. Biomarkers that identify clinical risk need to continue to be combined and administered in prospective studies that assess their predictive power. The underlying mechanisms driving development of the biomarker will then need to be elucidated in preclinical or *in vitro* models of disease. Only once the predictive framework is established, and mechanisms understood, will new therapeutic models and targets emerge for testing in clinical trials.

**Figure 1:**
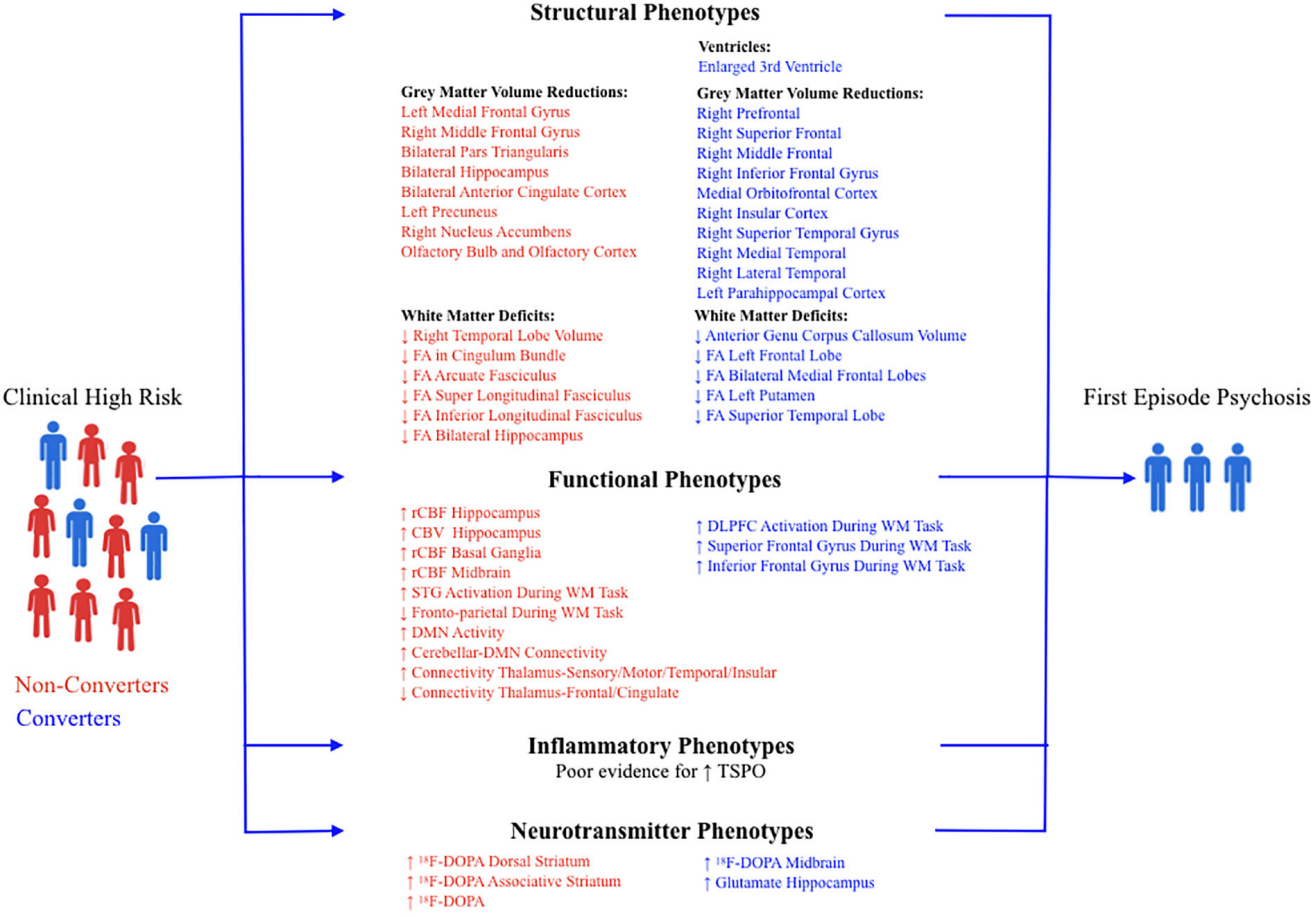
Summary of neuroimaging findings in non-converters (red) and converters (blue) at clinical high risk for psychosis. FA, Fractional Anisotropy; rCBF, Regional Cerebral Blood Flow; CBV, Cerebral Blood Volume; DMN, Default Mode Network; DLPFC, Dorsolateral Prefrontal Cortex; STG, Superior Temporal Gyrus; WM, Working Memory.

Neuroimaging has been successful in identifying multiple indicators of pathology in CHR subjects; some that represent generalized mental illness and are present in both converters and non-converters, and some that represent risk for psychosis and are present only in converters (see **Figure 1**). Structural MRI studies have identified multiple phenotypes in CHR subjects that convert to psychosis. Although enlarged lateral ventricles are well replicated in both first episode psychosis and chronic disease, only 3^rd^ ventricular expansion has been reported and replicated in CHR convertors vs non-convertors. As enlarged lateral ventricles are thought to be secondary to decreased grey matter volume, an enlarged 3^rd^ ventricle may represent earlier deficits in subcortical thalamic regions, or even the temporal lobes. However, although decreased thalamic volume has been reported in chronic disease, a recent study found no difference in thalamic volume in CHR subjects compared with controls (132). Furthermore, a longitudinal analysis of neuroimaging data from CHR subjects who later developed psychosis concluded that ventricular expansion was linked in time to progressive grey matter loss and not to structural changes in subcortical regions (133). Reductions in grey matter volume have been consistently reported in the frontal (superior frontal, prefrontal, middle frontal, medial orbitofrontal, inferior frontal gyri, and insular cortex) and temporal lobes (lateral temporal, medial temporal, and parahippocampal cortex) in CHR subjects that convert. Very interestingly, the degree and timing of grey matter loss may depend on age of symptom onset. In a recent report, Chung et al. evaluated baseline MRI parameters of converters and non-converters and observed that younger CHR subjects (12–17 years old) that converted to psychosis exhibited decreased grey matter volume at baseline and a less steep grey matter decline at first episode psychosis (134). However, older CHR subjects (> 18yrs old) that converted to psychosis did not have decreased grey matter volume at baseline, but exhibited a much steeper rate of volume loss as illness progressed. The first type is more insidious and ultimately debilitating and indicates that there is heterogeneity in the progression of grey matter loss among CHR subjects that convert.

Two other structural phenotypes warrant further exploration in CHR subjects, cerebral asymmetry and olfactory bulb volume loss. Reduced cerebral asymmetry is a common observation in established schizophrenia (135), and is more pronounced in the language areas of the temporal lobes and the pars triangularis and pars orbitalis in the inferior frontal gyrus. In healthy people, this asymmetry is thought to be related to maturation of language regions and the establishment of language dominance in one side of the brain. For example, verbal fluency is correlated with the degree of lateralization, and it’s been well established that patients with schizophrenia have decreased verbal fluency (136). CHR subjects appear to have reduced cerebral asymmetry, similar to schizophrenia, compared with controls (137). However, this warrants further exploration in subjects who convert. Abnormalities in the olfactory system have been reported in CHR subjects (138). Bilateral reductions in olfactory bulb volume in males, as well as reduced left olfactory grey matter volume, were observed in subjects at baseline. Furthermore, left olfactory bulb volume correlated with negative symptom severity. However, these phenotypes have not been compared between converters and non-converters.

White matter abnormalities are also present in CHR subjects, and they mostly overlap with implicated regions of grey matter reduction, i.e. the frontal and temporal lobes. Deficits reported are either reduced volume or reduced structural integrity as measured by diffusion tensor imaging. Of particular interest is that subjects who converted exhibited decreased thickness in the anterior genu of the corpus callosum, implying that its inclusion in prediction algorithms may improve accuracy.

Functional imaging has revealed several highly replicable findings in CHR subjects who convert to psychosis. Hippocampal hyperactivity and reduced recruitment during relevant cognitive tasks have been reported multiple times using different modalities including rCBF, CBV, and measurement of glutamate. Elevated activity is thought to result from an imbalance in excitation/inhibition secondary to interneuron dysfunction and may be responsible for the mesolimbic hyperdopaminergic state seen in patients, as evidenced by preclinical models. Functional dysconnectivity has been reported between multiple brain regions in CHR subjects at baseline, including increased activation in the amygdala and decreased activation in the ventrolateral prefrontal cortex during emotion labeling tasks and inappropriate activation of the superior temporal gyrus and lack of decoupling with middle frontal gyrus during verbal working memory tasks. Increased activation of the default mode network (DMN) at baseline, with decreased suppression during cognitive tasks, has been observed in CHR subjects. Subjects that convert exhibit abnormal thalamocortical connectivity, specifically hypoconnectivity between the thalamus and the prefrontal cortex and cerebellum, and hyperconnectivity between the thalamus and the sensory motor areas.

Inflammation has been strongly implicated in the pathophysiology of schizophrenia, in both FEP and chronic disease. Surprisingly, most studies have failed to find an increase in TSPO signaling in CHR subjects, either using 1^st^ or 2^nd^ generation radioligands. This may be due to the inference of TSPO as a marker for microglial activation, as it is known to be expressed on both microglia and astrocytes. Furthermore, the early evidence for an elevated signal used the 1^st^ generation radioligand, [^11^C]PK11195, which was later shown to have significant non-specific binding. Given the preponderance of evidence that inflammation is present during both the prodromal period and first episode psychosis, the lack of TSPO abnormalities may reflect more on the method than the pathophysiology. Furthermore, to the best of our knowledge there are no reports comparing TSPO signal between converters and non-converters, and these studies may help determine whether TSPO should be used moving forward or not.

Finally, CHR subjects that convert to psychosis have been shown to exhibit neurotransmitter abnormalities, including increased dopamine synthesis capacity in the dorsal and associative striatum. Higher levels predicted transition to psychosis, as did increased dopamine synthesis capacity in the midbrain. Furthermore, when given a verbal encoding and recognition task, CHR subjects showed a positive correlation between medial temporal lobe activation and striatal dopamine synthesis during encoding but not recognition.

One of the major challenges in using clinical or neuroimaging phenotypes discovered in CHR subjects is applying that knowledge at the individual level to predict conversion. The latest front in the prediction of psychosis is to apply machine learning methods to datasets of those phenotypes. Training algorithms on datasets of known outcome has allowed investigators to begin fine-tuning accuracies of prediction to greater and greater degrees. Seemingly, the greatest progress has come when combining modalities, such as clinical and neuroimaging, implying that heterogeneity within each modality may prevent anyone from being singularly adequate for prediction. One can hypothesize, then, that further combinations of modalities may finally allow for balanced accuracies to cross the 90^th^ percentile. Therefore, along with the known clinical and neuroimaging predictors, adding in peripheral blood phenotypes may aid as well. For example, Perkins et al. looked at peripheral blood analytes, specifically 15 analytes reflecting markers of inflammation, oxidative stress, hormones and metabolism, and were able to distinguish CHR converters from non-converters, with an area under the ROC curve of 0.88 (78). Furthermore, CHR subjects were found to have higher blood cortisol levels compared to controls, which moderately correlated with symptom severity, with higher baseline cortisol in those who converted (139).

Multiple challenges exist when using machine learning to create prediction algorithms. One major challenge is the small sample sizes of CHR populations, especially considering the low conversion rate. In order to attain large enough sample sizes, multi-site studies are necessary. However, multi-site studies incur their own challenges, most significant of which is inter-site variability in data collection and processing. Multiple strategies have been implemented to try to overcome this variability. One such strategy is the leave-one-out strategy, whereby an algorithm is trained on datasets from all sites but one, which is then used to validate the algorithm. Another is strategy is the healthy traveler design, in which healthy volunteers physically travel to each site in the study for scanner and software calibration. Furthermore, data must be collected on the same model equipment and must be processed using the same software. Software updates must be implemented at the same time across sites. Finally, overfitting of the model, due to small sample sizes, may explain some of the difficulties in validating external datasets and may also explain why accuracies appear to decrease with increasing sample size. It has been suggested that limiting the number of predictors compared to the number of converters may assist in solving this problem (119). One example of a large multi-site consortium trying to overcome these issues is the PSYSCAN Consortium (140). They have developed a protocol which aims to use multimodal methodologies (clinical, cognitive, genetics, blood, and imaging) and machine learning to create algorithms that predict conversion.

In conclusion, neuroimaging has significantly contributed to our understanding of developing abnormalities in the clinically high-risk population for psychosis. Further longitudinal research, in order to identify differences between converters and non-converters, large multi-site studies, the combination of multi-modal predictors, and machine learning algorithms that allow for prediction at the individual level will be necessary to identify the pre-conversion changes that are most clinically relevant and build more accurate prediction algorithms.

## Author Contributions

DG and JE equally contributed to the literature review, synthesis, and writing of the manuscript. All authors contributed to the article and approved the submitted version.

## Funding

This review has been supported by the National Institute of Mental Health (K23 MH114037 to DG. JE is supported by an R25 MH101079).

## Conflict of Interest

The authors declare that the research was conducted in the absence of any commercial or financial relationships that could be construed as a potential conflict of interest.
